# Congenital Heart Disease among Children Undergoing Echocardiography in the Department of Pediatrics of Tertiary Care Centre: A Descriptive Cross-sectional Study

**DOI:** 10.31729/jnma.8557

**Published:** 2024-04-30

**Authors:** Romila Chimoriya, Ritesh Chimoriya, Mandira Shrestha, Sabina Shrestha, Kailash Shah, Lopsang Lama, Kritika Rana

**Affiliations:** 1 Department of Pediatrics, Nepal Medical College Teaching Hospital, Jorpati, Kathmandu, Nepal; 2 School of Medicine, Western Sydney University, Campbelltown, Australia; 3 Translational Health Research Institute, Western Sydney University, Campbelltown, Australia

**Keywords:** *congenital heart disease*, *cross-sectional study*, *echocardiography*, *pediatrics*

## Abstract

**Introduction:**

Congenital heart disease in children are a major cause of infant mortality and morbidity. It is important to detect and manage these disorders timely as they are preventable. The objective of this study was to find out proportion of congenital heart disease in children in pediatric department in a tertiary hospital.

**Methods:**

This is a descriptive cross-sectional study carried out in the Department of Pediatric at Nepal Medical College and Teaching Hospital where all children (0-18 years) suspected to have congenital heart disease who underwent echocardiography were studied over a period of 1 year (2020-2021). The presence or absence of congenital heart disease were confirmed by echocardiography performed by pediatric cardiologist. The socioeconomic variables,clinical features and echocardiography findings were noted.

**Results:**

Out of total 249 patients,the proportion of patients diagnosed to have cardiac disorders was 73 with male predominance of 165 (66.26%). The most common age group was found to be neonates 111 (44%). The notable clinical features were murmur 47 (18.87%), tachypnoea 27 (10.84%) ,tachycardia 27 (10.84%) and cyanosis 9 (3.61%), clubbing 2 (0.80%), oedema 1 (0.40%), hypertension 9 (3.65%), murmur 47 (18.87%). Out of the total, there were 49 (19.67%) cases of acyanotic congenital heart disease, and 27 (10.84%) cases of cyanotic congenital heart disease.

**Conclusions:**

Our study focuses on early recognition of cardiac diseases which is crucial for preventing morbidity and mortality.

## INTRODUCTION

Congenital heart disease remains the most common birth defects with increasing prevalence of 9.41/1000 live births(8.602-10.253/1000 live births).^1^ Twenty eight percent of all major congenital anomalies consists of heart defects.^2^ Furthermore, the prevalence of congenital heart disease are increasing with 1 million patients with simple lesions, and half that number each with moderate and complex lesions in developing countries with advanced medical care facilities.^3^ The incidence of moderate and severe forms of CHD is about 6/1,000 live births (19/1,000 live births if the potentially serious bicuspid aortic valve is included), and of all forms increases to 75/1,000 live births if tiny muscular VSDs present at birth and other trivial lesions are included.^4^

The aim of this study was to find out proportion of congenital heart disease in children undergoing echocardiography in tertiary care centre.

## METHODS

This is a descriptive cross-sectional study carried out in the Department of Pediatric at Nepal Medical College and Teaching Hospital. Ethical approval was attained from the Research and Institutional Review Committee (IRC) of Nepal Medical College and Teaching Hospital ((Reference number: 36-079/080). All the patients in the age group 0-18 years who had clinical suspicion of congenital heart disease attending outpatient as well as inpatient from September 2021 to August 2022 were included for the study. Data from 1 September 2021 to 30 August 2022 were collected as pediatric echocardiography service was started from September 2021 in our centre. All patients with clinical features like cyanosis, difficulty in breathing, increased precordial activity, failure to thrive, cough, recurrent respiratory tract infection, chest pain, exercise intolerance, increased blood pressure, murmur, arrhythmias were suspected to have cardiac disease and was advised for echocardiography.^5^ The presence or absence of congenital heart disease were confirmed by echocardiography performed by pediatric cardiologist. Echocardiography findings were classified into two broad categories: acyanotic heart diseases and cyanotic heart diseases. The data of all the patients with sociodemographic variables, clinical features and diagnosis were documented. Patients with hemodynamically instability was excluded from the study.

Data were entered and analysed using SPSS software. In the study, descriptive statistical methods were utilized, which primarily focused on frequency distribution and percentage calculations. Each clinical characteristic and CHD type was tallied to find the total number of cases, and these frequencies were then converted into percentages of the total sample to calculate the proportion and distribution of various clinical features and CHD types among the patients. Graphs were derived from those frequencies.

## RESULTS

The total number of patients undergoing echocardiography was 249, with 165 (66.26%) being male and 84 (33.74%) being female. Neonates comprised the most common age group among the patients 111 (44%). The clinical findings observed in cases of CHD. Notable findings include mumur in 47 (18.87%), and decreased SpO2 in 10 (4.01%).

**Table 1:** Clinical finding of CHD cases (n= 249).

Clinical Features	n (%)
Irregular rhythm	1 (0.40)
Tachypnea	27 (10.84)
Tachycardia	27 (10.84)
Bradycardia	1 (0.40)
Cyanosis	9 (3.61)
Clubbing	2 (0.80)
Oedema	1 (0.40)
Hypertension	9 (3.61)
Murmur	47 (18.87)
Hepatomegaly	6 (2.41)
Crepts	6 (2.41)
Decreased SpO2	10 (4.01)
Decreased blood pressure	5 (2.01)

**Figure 1.**
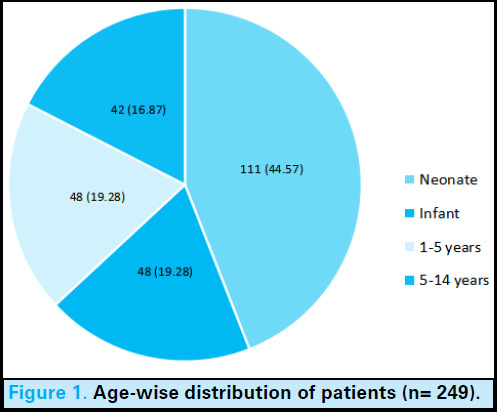
Age-wise distribution of patients (n= 249).

Congenital heart disease was found in 111 (44.57%) neonates (Figure 1).

Acyanotic heart diseases was found in 49 (19.67%) of the patients. Out of total cases, ventricular septal defect was found in 21 (8.43%) patients followed by atrial septal defect in 7 (6.83%), and post-operative acyanotic heart disease was found in 3 (1.21%) (Table 2).

**Table 2:** Types of acyanotic heart disease (n=249).

Clinical Features	n (%)
Ventricular septal defect	21 (8.43)
Atrial septal defect	17 (6.83)
Patent ductus arteriosus	6 (2.40)
Post-operative	3 (1.21)
Hypertrophic obstructive cardiomyopathy	1 (0.40)
Aortic stenosis	1 (0.40)
Total	49 (19.67)

There were 27 (10.84%) cases of cyanotic CHD. The pulmonary arterial hypertension 8 (3.21%), pulmonary stenosis 6 (2.41%), tetralogy of fallot 4 (1.60%), and persistent pulmonary hypertension 4 (1.60%), followed by double inlet left ventricle (DILV) 2 (0.80%). There was a single case (0.40%) each of Anomalous left coronary artery from the pulmonary artery (ALCAPA), Cor triatriatum, and postoperative cyanotic CHD (Table 3).

**Table 3:** Types of cyanotic heart disease (n= 249).

Clinical Features	n (%)
Tetralogy of fallot	4(1.61)
ALCAPA	1 (0.40)
Cor triatriatum	1 (0.40)
Pulmonary stenosis	6 (2.41)
Post-operative	1 (0.40)
Pulmonary arterial hypertension	8 (3.21)
Persistent pulmonary hypertension	4 (1.61)
DILV	2 (0.80)
Total	27 (10.84)

## DISCUSSION

Among 249 patients undergoing echocardiography, 70 cases of congenital heart disease was observed with proportion of 28%. The prevalence of a past study conducted in a similar setting showed the prevalence of congenital heart disease in Nepal was 0.7%.^6^ Indian studies had reported a wide variation in prevalence of CHD from 2.25 to 26 per 1000 live birth.^5,7,8^

In our study, there was a male predominance of 65.50%, a trend observed in similar studies.^7^'^9^ This gender disparity could be attributed to societal factors in our country, possibly stemming from a higher health' seeking behavior for male children among parents.^10^ Neonates (44%) constituted the most common age group in our study, which differed from other studies.^5^ This bias could be attributed to a substantial number of referrals from our neonatal intensive care unit.

The observed clinical features included irregular rhythm (0.40%), tachypnea (10.70%), tachycardia (10.70%), bradycardia (0.40%), cyanosis (3.60%), clubbing (0.80%), edema (0.40%), hypertension (3.60%), murmur (18.70%), hepatomegaly (2.40%), crepitations (2.40%), decreased oxygen saturation (SpO2) (4%), increased blood pressure (2%), decreased blood pressure (2%), and increased respiratory rate (25.40%). Similar studies showed clinical features of murmur (84.80%), tachycardia (41.50%), and tachypnea (36.30%) to be major clinical features, consistent with our study.^8^ Respiratory distress was found in 51.8% in neonates with CHD.^10^

In our study, cardiac diseases constituted over two-thirds of total cases, possibly due to enrolling all patients with clinical suspicions of cardiac diseases.

The prevalent cyanotic CHDs at our centre were PAH (3.2%), pulmonary stenosis (2.4%), and TOF (2%), a trend not in line with other studies. The disparity in the spectrum of cyanotic heart diseases could be attributed to the direct referral of most cyanotic cases to cardiac centres rather than our facility. Additionally, since our study population primarily consisted of neonates, the diagnosis of persistent pulmonary hypertension was made in four cases.

However, a limitation of our study was its singlecentre nature with a limited sample size. Additionally, most critically ill cardiac cases were directly referred to cardiac centres, potentially explaining the lower occurrence of complex cardiac diseases at our facility.

## CONCLUSIONS

Status of cardiac disease in our country is still iceberg as many children succumb to death prior to diagnosis of heart disease due to limited cardiac centres, lack of trained manpower and financial constraints. Any children with clinical suspicion of cardiac disease should be evaluated and diagnosis need to be ascertained by diagnostic modalities available so that mortality and morbidity can be reduced.
