# Erratum: Congenital Heart Disease among Children Undergoing Echocardiography in the Department of Pediatrics of Tertiary Care Centre: A Descriptive Cross-sectional Study

**DOI:** 10.31729/jnma.8679

**Published:** 2024-07-31

The online version of the article "Congenital Heart Disease among Children Undergoing Echocardiography in the Department of Pediatrics of a Tertiary Care Centre: A Descriptive Cross-sectional Study"^1^ has been updated according to the letter to the editor and the respective authors' reply published in JNMA Issue 275.

Due to corrections in the total number of patients undergoing echocardiography, minor corrections in the reported percentages, especially in the values after the decimal place, have been made.

Corrections have been made in the results section of the abstract, Figure 1, results, and the discussion. The corrected sections are as follows:

In the results section of the abstract, "165 (66.26%) male and 84 (33.74%) female, neonates 111 (44.57%), murmur 47 (18.87%), tachypnea 27 (10.84%), tachycardia 27 (10.84%), decreased oxygen saturation 10 (4.01%)" has been corrected.

In the results section of the manuscript, "percentages of neonates 111 (44.57%)" in the first paragraph, "atrial septal defect 17 (6.83%)" in the second paragraph, and "tetralogy of fallot 4 (1.61%), and persistent pulmonary hypertension 4 (1.61%)" in the third paragraph have been corrected.

In the discussion section, "Among 249 patients undergoing echocardiography, 49 (19.67%) cases of acyanotic CHD and 27 (10.84%) cases of cyanotic CHD were observed" in the first paragraph, "male predominance of 165
